# MEDICAL LABORATORY SCIENTISTS’ ROLE IN THE CARE OF AN AGING POPULATION

**DOI:** 10.1093/geroni/igad104.1691

**Published:** 2023-12-21

**Authors:** Tripat Kaur

**Affiliations:** Hillsborough Community College, Tampa, Florida, United States

## Abstract

Medical Laboratory Scientists (MLS) are a group of highly skilled professionals in the healthcare industry who are responsible for working on specimens and providing results that are accurate in diagnosing medical disorders and monitoring treatments. These heroes work behind the scenes and face a critical shortage of personnel required to meet the needs of an ageing population. The number of clinical tests has increased 388% since 1993, from 12,683 to nearly 62,000. In addition, the Centers for Disease Control reports that for the >500 million primary care patient visits each year, one out of three patient encounters result in the ordering of at least one laboratory test. To meet these high testing volumes medical and clinical laboratories are highly automated with instrumentation utilized in all aspects of testing to improve the turnaround time for reporting results. Programs that offer medical laboratory science programs often must prepare the students for this highly technical field. To assist in the shortages that the laboratories faced, 6 years ago, BayCare Health System approached Hillsborough Community College Health Science division about setting up a post-baccalaureate medical laboratory science program. The program has now had approximately 100 graduates who have jobs around the Tampa Bay hospitals. Educational institutions face challenges in meeting current needs because of the lack of visibility of the medical laboratory science profession, a drop in the total enrollment in colleges over the last 5 years, and a rise in the national median age of the current population.

